# Report of the Proceedings of the Medical Association of the State of Georgia, at Its Annual Meeting Held in the City of Atlanta, April 13th and, 14th, 1859

**Published:** 1859-06

**Authors:** A. G. Thomas

**Affiliations:** Sec. of The Med. Asso’n of Ga.


ATLANTA
### Medical and Surgical Journal.

VOL. IV.] JUNE, 1859. [No. X.

### ORIGINAL COMMUNICATIONS.

### ARTICLE I.

*Report of the Proceedings of the Medical Association of the State of Georgia, at its Annual Meeting held in the City of Atlanta, April* 13*th and* 14*th*, 1859.

Pursuant to adjournment the Medical Society of the State of Georgia assembled in the City Hall, Atlanta, at 11 o'clock on the morning of the 13th of April, 1859.

The Society was called to order by the President, Dr. Joseph P. Logan of Atlanta, and the deliberations opened with prayer by Rev. Dr. Wilson of Atlanta.

The Recording Secretary being absent, on motion of Dr. H. F. Campbell, of Augusta, Dr. W. S. Meiére of Madison, was requested to act as Secretary *pro tem.*

The roll being called by the Secretary, the following members responded to their names: S. W. Burney, of Forsyth, A. Means, Henry Gaither, of Oxford, Samuel B. Clarke, of Richmond Factory, Richmond Co., J. G. Westmoreland, J. N. Simmons, H. W. Brown, Jno. W. Jones, Thos S. Powell, B. M. Smith, Jas. F. Alexander, M. H. Oliver, J. M. Boring, Hayden Coe, T. C. H. Wilson, V. H. Taliaferro, of Atlanta, A. A. Bell, Maxey's, E. J. Roach, Longstreet, Pulaski Co., T. J. Barkwell Hawkinsvlle, L. A. Dugas, H. F. Campbell, Augusta, L. P. Green John T.

Banks Zebulon, S. H. Dean, Connally, Griffin, A. M, Boyd, Cave Spring, A. M. Parker Salt Spring, Campbell Co., W. S. Meiére, Madison.

Dr. Campbell, of Augusta, then presented to the Society, for distribution among its members, a supplement to the Southern Medical & Surgical Journal, containing, besides very interesting editorial and select matter, the History, Constitution and By-Laws of the Society.

On motion of Dr. Coe, the Constitution and By-Laws of the Society were read by the Secretary. The proceedings of the last annual Meeting held in Madison were then read and confirmed.

On written application the following gentlemen were duly elected members of the Society: Drs. E. O. Ware, Robert. Battey, T. J. Word, Rome, W. A. Culbertson, Cave Spring, J. A. Stewart, Conyers, A. S. Whitaker, Jonesboro, J. R. McAfee, Dalton, R. G. W. Maffitt, Dalton, W. A. Shelby, W. V. Aderhold, G. G. Crawford, H. Westmoreland, J. Gilbert, G. W. Humphries, J. D. Boyd, J. L. Hambleton, A. G. Thomas, W. P. Hardin, R. J. Massey, J. M. Sessions, N. D'Alvigny, D. O C. Heery, B. O. Jones, B. F. Bomar, Atlanta, C. A. McKinley, G. L. Hudson, Newnan, A. M. Moore, J. C. Avery, Decatur, G L. Jones, G. L. Johnson, Palmetto, J. T. Slaughter, Villa Rica, G. W. Pitts, Star, Butts Co., B. L. Jones, Savannah. B. F. Hodnett, Rough & Ready, W. D. Cunningham, Jasper Pickens Co., L. S. Cunningham, Big Creek, Forsyth Co., E. Griffin, Rough & Ready, W. H. Watkins, Franklin F. O. Donnelly, Greenville, N. B. Drewry, Sharon Grove, Fayette Co., S. P. Taylor, Haralson, Johnson Matthews, Yellow River, S. W. Leak, J. F. Donehoo, Fayetteville, W. C. Smith, Grantville, J. Walker, Longstreet, M. T. Fort, T. D. L. Ryan, J. H. Vakley, Hawkinsville, Joseph Jones, Augusta, J. K. Mitchell, Lawrenceville, D. A. Matthews, Millstone, George Lumpkin, Stephens, W. Moody, Maxey's, Samuel P. Lumpkin, Watkinsville, W. P. Bond, Lithonia, R. A. T. Ridley, LaGrange, W. A. Spier, Grantville, A. P. Brown, Cumming, T. Hackenhull, Dawsonville, S.

Malone, Fairburn, C. W. Smith, Jonesboro, W. F. Thomason, Sugar Valley, D. H. Payne, Marietta, H. S. Davenport, ———, B. M. Tidwell, Fairburn, F. M. Brantley, Merriwether, D. G. Hunt, Calhoun, L. H. Jordon, Coloparchee, D. M. Williams, Griffin.

After the enrolling of the names of new members, the Society adjourned till 3 o'clock, p. m.

AFTERNOON SESSION.

The Society was called to order at 3 o'clock by the President.

The election of officers being in order, the President ordered a ballot for President, Dr. Banks proposed the name of Dr. F. S. Colley. Dr. Alexander proposed the name of Dr. L. A. Dugas. Dr. Barkwell proposed the name of Dr. H. F. Campbell. Before the ballot Drs. Dugas and Campbell withdrew their names. Society proceeded to ballot for President—on counting the ballot it was found that Doctor Colley had received all the votes cast, Dr. Colley was therefore declared unanimously elected.

After ballot for 1st Vice President, Dr. R. A. T. Ridley was declared duly elected. By ballot Dr. H. Coe was elected 2d Vice President. By ballot Dr. A. G. Thomas was elected Recording and Corresponding Secretary and Treasurer.

On motion the following committee was appointed to induct the President elect into the chair: Drs. Campbell, Alexander and Battey. Dr. Logan in retiring from the chair delivered an appropriate and felicitous address, full of interesting statements and valuable suggestions.

On motion of Dr. Banks—the rules being suspended—Dr. B. L. Jones introduced the following resolution, which was unanimously adopted: "*Resolved*, That the thanks of this body be tendered to Dr. Jos. P. Logan for the faithful, efficient and impartial manner in which he has discharged the duties of President during his term of office, and also for the pertinent and truthful address to which we have just listened."

On motion of Dr. Burney the following committee was appointed to recommend the names of such members of the Society, as the committee should select, as delegates to the American Medical Association: Drs. Logan, Battey, Roach, Jos. Jones, Boyd, Taylor and Burney.

Rules being suspended, on motion of Dr. Means, Dr. Wm. T. Grant, of Thompson, was allowed to withdraw his name from the roll of members of this Society. After which, on motion of Dr. Roach, Society adjourned till 10 o'clock Thursday morning.

---

THURSDAY, April 14th, 1859,

10 o'clock, a. m.

Society called to order by the President. Roll called; minutes of the preceding day read and approved. Rules being suspended the following resolution was offered by Dr. J. G. Westmoreland and adopted: "*Resolved*, That Dr. R. L. Bozeman, of Ala., be invited to a seat with the Society to-day."

Rules being further suspended, Dr. Burney moved to appoint a committee of three to wait upon Dr. Logan and request a copy of his address for publication: motion carried. The President appointed as that committee, Drs. Burney, Coe and Dean.

Dr. Means, after a few appropriate remarks, introduced the following resolution: "*Resolved*, That the name of this Society be altered from the Medical Society of the State of Georgia, to the Medical Association of the State of Georgia." Resolution unanimously adopted.

Reports from Auxiliary Societies being called for, Doctor Banks reported that an Auxiliary Society had been established in Griffin according to the Constitution and By-Laws of the State Association; that the Society was composed of twelve members, and was increasing; he hoped that the Auxiliary Medical Society of Griffin would be acknowledged by the Association. Dr. Campbell moved that the report be received, and the Society acknowledged, which motion was carried.

Correspondence called for—no report. Call for written communications. Dr. Juriah Harris, of Savannah, through Dr. J. G. Westmoreland offered an apology for not present- his Ess'y which he had been appointed to prepare, stating that unavoidable circumstances had prevented him from finishing it. On motion of Dr. Means, Dr. Harris' apology was received, and he was requested to prepare the Essay for the next meeting of the Association.

Dr. Campbell, of Augusta, presented the following, entitled: "An Essay on Chclera Infantum," by H. W. Dessausure Ford, M. D., Prosector to the Prof. of Surgery in the Medical College of Georgia, and rendered an apology for Dr. Ford for his failure to present the Essay. On motion, Dr. Ford was requested to have his Essay published in one of the medical journals as written for the Association. Rules being suspended, Dr. Meiere offered the following resolution, which was carried: "*Resolved*, That the President appoint a committee of five to revise the Constitution and By-Laws of the Association, and report at next meeting." The President appointed as that committee, Doctors Meiere, Banks, Oliver, Battey and H. F. Campbell. Rules further suspended. The committee on Delegation to the American Medical Association, reported the following names of members of this body as suitable delegates: Drs. West and Sullivan, of Savannah; Doughty and Robert Campbell, of Augusta; Nottingham and Boon, of Macon; Means, of Oxford; Alexander, of Atlanta; Stanford and Flewellen, of Columbus; McClesky and Carleton, of Athens; Roach, of Pulaski county; Stevens, of Albany; Hillyer and Battey, of Rome; Dannielly, of Merriwether; Burney, of Forsyth , McAfee, of Dalton; Ridley, of LaGrange; Banks, of Pike; Brown of Cumming, and Meiere, of Madison.—On motion of Dr. Campbell, the report of the committee was received and adopted. Dr. Banks offered the following resolution, which was adopted: "*Resolved*, That the delegates to the American Medical Association, this day appointed, be authorized to select alternates from the members of this Society, in case of the inability of any of them to attend the meeting of said American Medical Association." Dr. Campbell then presented the following title of a paper, with the accompanying motto:

"An Essay on the adaptation of Climate to the Consumptive, for a permanent residence; embracing an Examination of the climate of certain localities of frequent resort; and also, an Investigation of the degree of adaptedness of the Pacific Climates of the United States.—Presented to the Medical Association of the State of Georgia, at its annual meeting, held at Atlanta, April 13, 1859. By Wm. Henry Doughty, M. D., of Augusta, Ga." Motto:

"As 'the possible is immense,' so the human mind if the legitimate object of all science, (which is to observe facts and to trace their relations and sequences) is kept steadily in view will be continually verging towards Truth in the investigation of physical causes."—FORRY.

He then stated that Dr. Doughty was unable to be present, and hoped to be excused; after which he read a portion of the able Essay of Dr. Doughty.

Dr. Campbell offered also, an abstract of an "Essay on *Quinine:* its therapeutical action being expended solely upon the *middle* or *fibrinous coat* of the BLOOD VESSELS, by which interpretation alone are its phenomena satisfactorily explicable.—By Robt. Campbell, M. D., Adjunct Prof. of Obstetrics and Dem. of Anat. in the Medical College of Georgia."

On motion, the Association adjourned until 2½ o'clock, p. m.

AFTERNOON SESSION.

Association called to order at 2½ o'clock. Regular business being continued, Dr. Jos. Jones presented a "Report of a case of Fracture of Neck of the Scapsula;" also, a "Report of a case of Aneurism in the gluteal region."—L. A. Dugas, M. D., Prof of the Prin. and Prac. of Surgery in the Medical College of Georgia." He also presented an Essay on "The Changes of the Blood in Malarial Fevers.—By Jos. Jones, M.D., Prof. of Chemistry and Pharmacy in

Medical Col. of Georgia." An abstract of a "Report of fifteen cases of Lithotomy," with the calculi acompanying, was presented by H. F. Campbell, M. D., Prof. of Anatomy in Med. Col. of Georgia. An Essay on "Puerperal Fever" was then read by Dr. Dean, of Conyers.

An Essay entitled "Quackery and its Cure," was then read by Dr. A. G. Thomas, of Atlanta. A "Report of a case of Vesico Vaginal Fistula," was presented by Dr. Battey of Rome. An Essay on "The Pathology of Phlegmasia Dolens," was then read by Dr. H. Coe, of Atlanta. Dr. Taliaferro, Professor of Materia Medica in Oglethorpe Med. College, offered an apology for not presenting his Essay, stating he had begun to prepare it, but was unable to complete it; subject: "Phthisis Pulmonalis, its causes and treatment." Dr. W. F Westmoreland, Prof. of Surgery in Atlanta Medical College, offered an excuse for not presenting his Essay, it not being in his power to finish it; subject: Pyemia.

Rules being suspended, Dr. J. P. Logan moved that a committee be appointed to select Essayists for the next meeting. Motion amended by adding: "and to recommend place for next meeting." Motion carried—Committee appointed by the President: Drs. Logan, Campbell and Word.

Oral communications called for; no report. Rules being suspended, Dr. Means, on behalf of the Faculty of Atlanta Medical College, invited the members of the Association to visit their College building.

New business being called, Dr. Battey introduced the following resolution, which was adopted: "*Resolved*, That a committee of three be appointed by the Chair to report at our next meeting upon the evidences of advancement in medical science, as exhibited in the literary productions of the medical men of Georgia."

The President appointed as that committee the following gentlemen: Drs. Logan, Battey and Sullivan.

Dr. Banks offered the following resolution: "*Resolved*, That the Chair appoint a committee of three members of the Association, to which committee the Essays and Reports of the Association be referred for publication; the same to be published in connection with the other proceedings of the Association in the form of a report."

A motion to reconsider Dr. Banks' resolution was made and carried. Dr. Battey offered in lieu of Dr. Banks' resolution the following: "*Resolved*, That the Essayists be authorized to present their Essays to any of the medical journals of the State, which they might select, for publication."—Resolution was adopted. On motion of Dr. Meiere, the Association proceeded to ballot for orator at the next meeting. On counting the ballot, Dr. H. W. D. Ford, of Augusta, was declared elected. Dr. Banks was elected as alternate.

Committee on place of next meeting, recommended Rome. Report of Committee adopted.

Dr. Campbell then proposed that the Southern Medical and Surgical Journal be no longer considered the exclusive organ of the Association and that all the Medical Publications of the State be requested to publish the proceedings. Proposition of Dr. Campbell accepted. Dr. Word offered the following resolution which was adopted: "*Resolved*. That the thanks of this body be tendered to the editors of the Southern Medical and Surgical Journal, the late organ of the Association, for their kindness done this body in publishing for several years its transactions without cost or charge. Also, to Dr. H. F. Campbell, for the courteous proposition made at this meeting, to extend to the other medical journals of the State equal participancy in all the publications of the Association in the future."

The President appointed a committee of arrangements for next meeting, Drs. Battey, T. J. Word, Hillyer, Miller and R. C. Word of Rome.

Dr. Clark of Richmond offered the following resolution which was adopted:—"*Resolved*, That we, the members of the Medical Association of the State of Georgia, tender to our medical brethern of the city of Atlanta our heartfelt thanks for their very generous hospitalities and warm reception in their city, and that we will ever hold it in grateful remembrance, and that it will form a most pleasant episode in the history of our professional lives. "*Resolved*, that our

Secretary be requested to have these resolutions published in the city papers."

There being no further business before the Association, on motion the Association adjourned to meet in the City of Rome on the 2d Wednesday in April, 1860.

A. G. THOMAS, M. D.,
Sec. of the Med. Asso'n of Ga.

